# Elevated dietary magnesium during pregnancy and postnatal life prevents ectopic mineralization in *Enpp1^asj^* mice, a model for generalized arterial calcification of infancy

**DOI:** 10.18632/oncotarget.16687

**Published:** 2017-03-29

**Authors:** Joshua Kingman, Jouni Uitto, Qiaoli Li

**Affiliations:** ^1^ Department of Dermatology and Cutaneous Biology, The Sidney Kimmel Medical College, and the PXE International Center of Excellence in Research and Clinical Care, Thomas Jefferson University, Philadelphia, PA, USA

**Keywords:** mouse model, ectopic mineralization, maternal diet, magnesium, generalized arterial calcification of infancy, Pathology Section

## Abstract

Generalized arterial calcification of infancy (GACI) is an autosomal recessive disorder caused by mutations in the *ENPP1* gene. It is characterized by mineralization of the arterial blood vessels, often diagnosed prenatally, and associated with death in early childhood. There is no effective treatment for this devastating disorder. We previously characterized the *Enpp1^asj^*mutant mouse as a model of GACI, and we have now explored the effect of elevated dietary magnesium (five-fold) in pregnant mothers and continuing for the first 14 weeks of postnatal life. The mothers were kept on either control diet or experimental diet supplemented with magnesium. Upon weaning at 4 weeks of age the pups were placed either on control diet or high magnesium diet. The degree of mineralization was assessed at 14 weeks of age by histopathology and a chemical calcium assay in muzzle skin, kidney and aorta. Mice placed on high magnesium diet showed little, if any, evidence of mineralization when their corresponding mothers were also placed on diet enriched with magnesium during pregnancy and nursing. The reduced ectopic mineralization in these mice was accompanied by increased calcium and magnesium content in the urine, suggesting that magnesium competes calcium-phosphate binding thereby preventing the mineral deposition. These results have implications for dietary management of pregnancies in which the fetus is suspected of having GACI. Moreover, augmenting a diet with high magnesium may be beneficial for other ectopic mineralization diseases, including nephrocalcinosis.

## INTRODUCTION

Ectopic mineralization, *i.e*., deposition of calcium and phosphate complexes in soft connective tissues, represents a broad class of diseases and a significant medical problem [1, 2]. These diseases include hardening of the arteries associated with aging, chronic kidney disease, osteoarthritis, cancer, diabetes, and autoimmune diseases. Ectopic mineralization, particularly when affecting the cardiovascular system, is a major cause of morbidity and mortality. For example, it has been demonstrated that coronary artery calcification in a cohort of 25,253 patients was an independent risk factor to death, the relative risk being up to 12.5-fold [1]. Vascular calcification is a common complication in calciphylaxis, a highly morbid disorder seen mostly in patients with chronic kidney disease [3]. There are two forms of pathological mineralization involving peripheral connective tissues. Metastatic calcification results from elevated serum levels of phosphate and/or calcium exceeding the homeostatic capacity of cells and tissues, in conditions such as chronic renal failure and hyperparathyroidism. In dystrophic calcification, serum calcium and/or phosphate levels are normal, but calcification is a secondary consequence of trauma to the tissues, for example in autoimmune diseases, such as systemic lupus erythematosus, scleroderma and dermatomyositis [4]. Dystrophic calcification is also frequently noted in diseased tissue, such as chronic ulcers and granulomas as well as in benign and malignant neoplasms. Collectively, ectopic mineralization is a consequence of a number of contributing genetic, metabolic, and environmental factors which has made uncovering the precise molecular basis and clinical management of these disorders exceedingly difficult.

A number of heritable ectopic mineralization disorders and their corresponding mouse models, as exemplified by generalized arterial calcification of infancy (GACI), provide insight into the pathophysiology of soft tissue mineralization under normal calcium and phosphate homeostasis [5, 6]. GACI is a rare heritable disease characterized by prenatal onset of widespread mineralization of large and medium-sized arteries, resulting in cardiovascular collapse and death in the neonatal period [7]. GACI is often diagnosed prenatally through ultrasound, and the newborns manifest with severe hypertension, cardiomyopathy and heart failure, resulting in demise of the affected individuals in most cases during the first year of life [8]. GACI is caused in most cases by loss-of-function mutations in the *ENPP1* gene, which codes for ectonucleotide pyrophosphatase/phosphodiesterase 1 (ENPP1), an extracellular membrane bound glycoprotein that hydrolyzes adenosine triphosphate into adenosine monophosphate and inorganic pyrophosphate (PPi) [9, 10]. In the absence of the ENPP1 activity, plasma levels of PPi, a powerful inhibitor of ectopic mineralization, are significantly reduced, and therefore, progressive vascular mineralization takes place. In addition, *ENPP1* mutations have been identified in some patients with pseudoxanthoma elasticum (PXE), another heritable ectopic mineralization disorder, although most cases with this disorder harbor mutations in the *ABCC6* gene [6, 11].

There is no effective or specific treatment for GACI. A few studies have suggested that administration of bisphosphonates, stable and non-hydrolyzable PPi analogues, might be helpful in counteracting the ectopic mineralization and reducing mortality in GACI [8, 12, 13]. However, a potential complication of this approach is the severe skeletal toxicity associated with prolonged use of bisphosphonates in patients with GACI [14]. Lack of consensus about the efficacy of these compounds and limited available data make it difficult to determine if bisphosphonates offer a safe and effective treatment for GACI.

A number of mouse models, both spontaneous and genetically engineered, have been described to recapitulate the clinical features of human GACI due to genetic alterations in the *ENPP1* gene. One of them, the *Enpp1^asj^* mouse (hereafter referred to as *asj*), was recently identified as a result of ENU treatment in The Jackson Laboratory Neuromutagenesis Program [15]. Pathological examination of these mice revealed a stiff posture, unbendable joints in the front legs with severe osteoarthritis and mineralization, and consequently, this mutant mouse was designated as “ages with stiffened joints (*asj*)”. These mice harbor a homozygous missense mutation in the *Enpp1* gene (p.V246D) that results in markedly reduced ENPP1 enzymatic activity and lowered plasma PPi concentration that subsequently allows for ectopic mineralization of soft connective tissues in the skin and arterial blood vessels to ensue [15]. Previous studies in *Abcc6^−/−^* mouse model of PXE revealed that an increase in dietary magnesium by five-fold over that in control diet completely abolished ectopic mineralization in the skin [16, 17]. In this study, we investigated the effects of dietary magnesium supplementation on ectopic mineralization in the skin and vascular tissues in *asj* mice, a model for GACI which shares genotypic and phenotypic overlap with PXE. Our results suggest that dietary magnesium supplementation, if administered during pregnancy and continue to postnatal period, may provide an effective way to counteract ectopic mineralization that causes considerable mortality in GACI.

## RESULTS

### High magnesium content in the diet prevents ectopic mineralization in the offspring

In this study, we tested the hypothesis that diet augmented with magnesium might counteract the ectopic mineralization in the skin and vascular tissues, using *asj* mice as a preclinical platform. Pregnant mothers were placed on either control diet or magnesium enriched diet during the entire pregnancy and the subsequent postpartum period. Wild type and *asj* mice at 4 weeks of age at weaning were placed on specific diets, either control diet or high magnesium diet for additional 10 weeks, as indicated in Table 1. At the age of 14 weeks, tissue mineralization was determined in the *asj* offspring.

**Table 1:** Experimental groups of *Enpp1^asj mice by genotype and treatment^1*)

Group	Genotype of pups	Diet pregnant mothers placed on	Diet pups placed on	No. of pups examined (M+F)
A	WT	control	control	9 (6+3)
B	asj	control	control	10 (5+5)
C	asj	control	High Mg	11 (6+5)
D	asj	High Mg	control	13 (6+7)
E	asj	High Mg	High Mg	12 (6+6)

^1)^ The pregnant mothers were placed on either control diet or magnesium-supplemented diet (high Mg diet) during pregnancy and nursing. The pups, at 4 weeks of weaning, were assigned to either control diet or high Mg diet for additional 10 weeks. The mice were sacrificed at the age of 14 weeks for mineralization analysis. WT, wild type; M, male; F, female.

**Figure 1:**
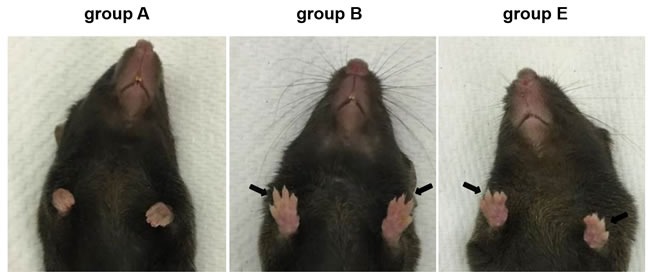
Magnesium treatment improves the stiffened joints phenotype in *asj* mice at 14 weeks of age The *asj* mice on control diet (group B) develop stiffening of the joints leading to contractures as shown on the front paws (arrows) in comparison with a corresponding wild type mouse (group A). The *asj* mice on high magnesium diet whose mothers were also placed on the same diet during pregnancy and nursing had improved phenotype of stiffening of the joints in the front paws (group E).

We have previously demonstrated that the *asj* mice on control diet develop stiffening of the joints, particularly the forepaws, which resulted in a slow, hobbling gait that worsened as they aged (Figure 1, group B), a finding that was not present wild type mice (Figure 1, group A) [15]. Among all groups that were tested, the *asj* mice fed high magnesium diet whose mothers were also fed high magnesium diet (Figure 1, group E) showed less stiffened phenotype of their forepaws when examined at 14 weeks of age. The mice were sacrificed, and muzzle skin, kidneys and aorta were harvested for mineralization analysis.

**Figure 2:**
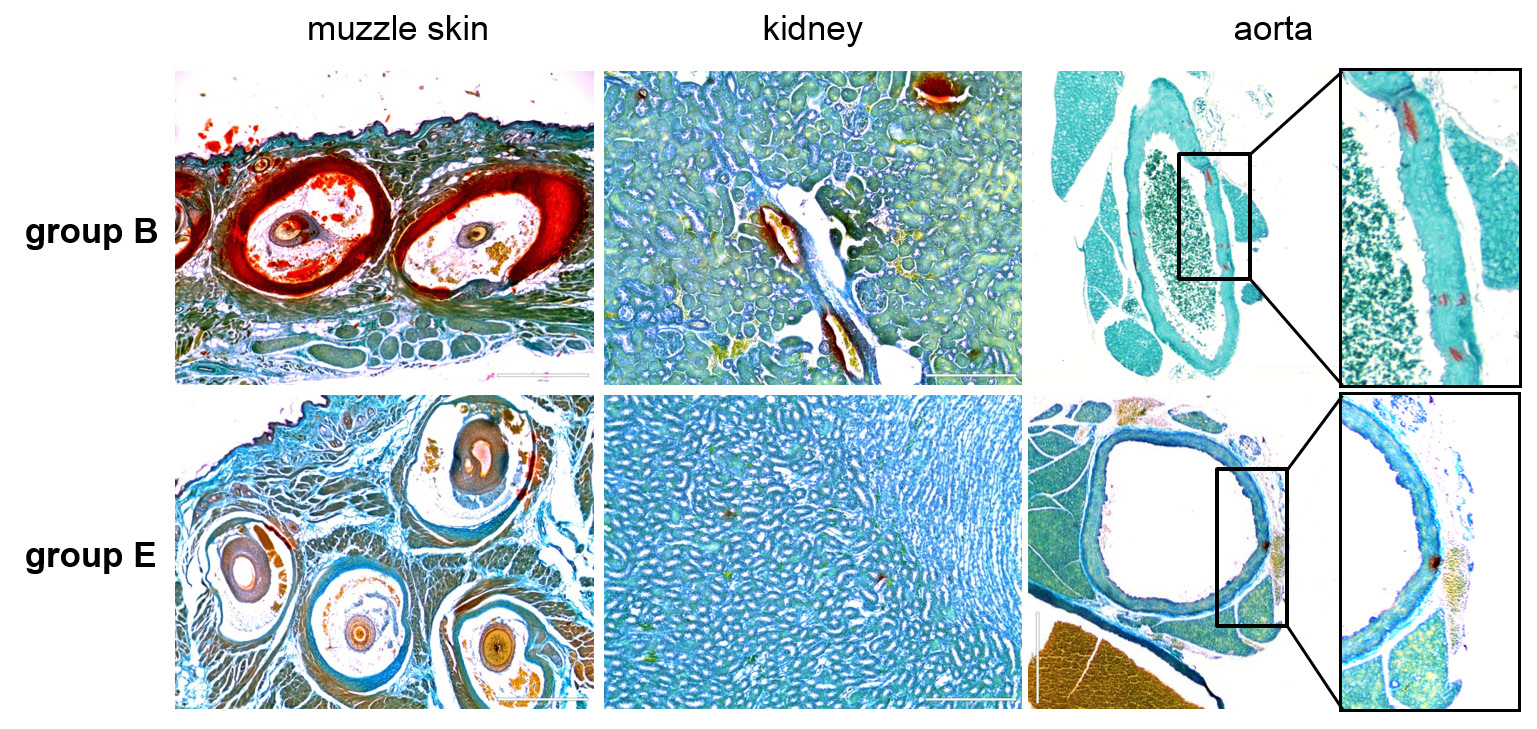
Magnesium treatment prevents ectopic soft tissue mineralization in *asj* mice as revealed by histopathology The *asj* mice and their respective mothers placed on control diet develop ectopic mineralization of the dermal sheath of vibrissae in muzzle skin, kidney and aorta, when examined at 14 weeks of age by histopathology with Alizarin red stain (group B). Markedly reduced mineral deposition was noted in *asj* mice fed high magnesium diet when their respective mothers were also placed on high magnesium diet during pregnancy and nursing (group E). Scale bar = 0.4 mm.

**Figure 3:**
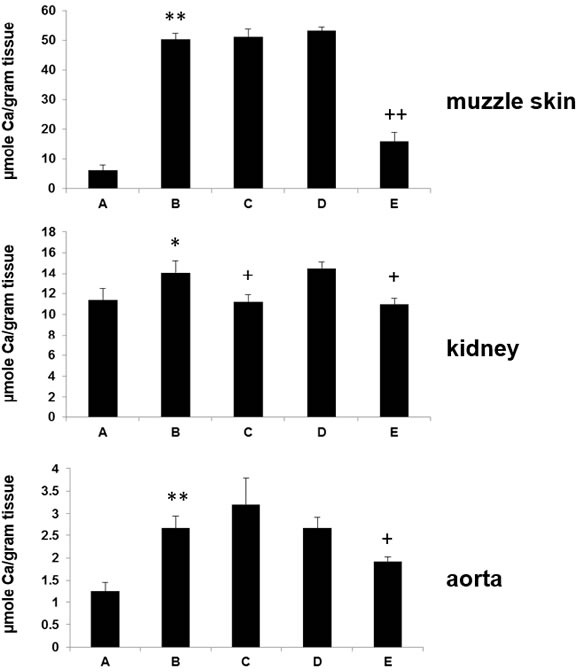
Magnesium treatment reduces ectopic soft tissue mineralization in *asj* mice as determined by the direct chemical assay of calcium Muzzle skin biopsies, kidney and aorta were harvested and calcium content was quantitated by a chemical assay. Note the significantly elevated calcium content in *asj* mice (group B) as compared with the wild type mice (group A) on the control diet. Treatment of *asj* mice with diet supplemented with magnesium whose pregnant mothers were also kept on the same diet (group E) resulted in a significant reduction in the calcium content in these tissues in comparison with the *asj* mice on control diet (group B). Feeding *asj* mice with magnesium after weaning whose mothers were fed control diet (group C) also significantly reduced the calcium content of the kidney as compared with *asj* mice on control diet (group B). Mean ± SE; *n* = 9-13 mice per group. * *P* < 0.05, ** *P* < 0.01, *vs* group A; ^+^ *P* < 0.05, ^++^ *P* < 0.01, *vs* group B.

The effect of diet enriched with magnesium on ectopic mineralization was assessed by two independent examinations. The left side muzzle skin, left kidney and descending thoracic aorta were collected and processed for semi-quantitative histopathological examinations. The degree of mineralization in right side muzzle skin, right kidney and abdominal aorta was quantified by a direct chemical assay of calcium content in solubilized tissues. Specifically, Alizarin red stains in wild type mice did not show any signs of mineralization (group A, data not shown), while extensive mineralization was noted in the dermal sheath of vibrissae in muzzle skin, kidney and aorta in *asj* mice on control diet whose mothers were also placed on control diet (Figure 2, group B). Elevated magnesium content in the maternal diet (five-fold) resulted in significantly reduced mineralization in *asj* offspring who were also placed on high magnesium diet (Figure 2, group E). The difference in tissue mineralization was also demonstrated by quantitative assay of calcium content in these tissues. High magnesium content in the diet of *asj* pups post weaning (Figure 3, group C) or in the maternal diet alone (Figure 3, group D) did not appreciably change the degree of mineralization in muzzle skin and aorta, as compared to the corresponding mice fed control diet (Figure 3, group B). In contrast, the *asj* mice fed a high magnesium diet with mothers on the same diet (Figure 3, group E) showed significantly reduced amount of calcium in muzzle skin, kidney and aorta, and quantitatively the calcium values were at the same low level as noted in wild type mice (Figure 3, group A). In addition, the *asj* mice fed a high magnesium diet whose mothers were on control diet also showed markedly reduced calcium content in the kidney (Figure 3, group C). Collectively, histopathologic analyses and quantitative calcium determinations demonstrated that elevated magnesium content (five-fold) in the diet prevents ectopic mineralization in *asj* offspring with mutations in the *Enpp1* gene.

**Figure 4:**
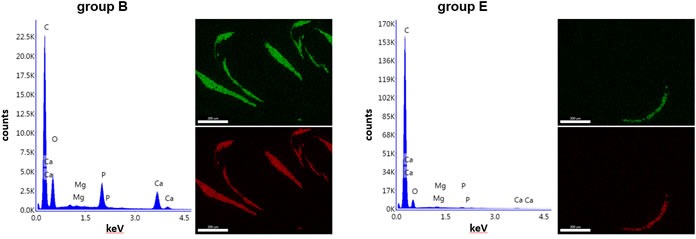
Energy dispersive X-ray analysis demonstrates hydroxyapatite in the muzzle skin biopsies containing mineral deposits in the dermal sheath of vibrissae Elemental composition analysis reveals the presence of calcium (Ca) and phosphorus (P) as the principal ions in the muzzle skin of *asj* mice in groups B and E. There is very little magnesium (Mg) present in the tissues. The presence of carbon (C) reflects the carbon carrier that holds the samples. X-ray topography of the distribution maps of calcium (green) and phosphorus (red) reveals co-localization in the areas of ectopic mineralization. Scale bar = 0.2 mm.

The magnesium content in the tissues was also assessed in *asj* mice as a result of feeding on magnesium-supplemented diet. The tissues from *asj* mice on control diet (group B) and high magnesium diet (group E) were analyzed at 14 weeks of age. First, the elemental composition in muzzle skin sections was analyzed by energy dispersive X-ray of the mineralized areas in the vibrissae. The analysis revealed calcium and phosphorus as the principal ions in the tissues (Figure 4). Topographic mapping revealed that these ions co-localize in the mineralized connective tissue capsule, suggesting the presence of hydroxyapatite. Magnesium does not constitute for the major element in the tissues, and in fact, as compared to calcium and phosphorus, magnesium signal is extremely low (Figure 4). Second, the amount of magnesium was quantitatively measured in solubilized muzzle skin, kidney and abdominal aorta samples. The amount of magnesium (μmol Mg/gram tissue) was not statistically different: 22.6 ± 0.9 and 21.6 ± 2.4 for muzzle skin, 15.3 ± 1.1 and 16.2 ± 1.3 for kidney, and 7.8 ± 0.4 and 7.6 ± 0.4 for abdominal aorta, from *asj* mice in groups B and E, respectively (mean ± SE).

### High magnesium content in the diet alters serum and urinary mineral profiles

To examine the metabolic consequences of the experimental diets, the serum and urine concentrations of calcium, phosphorus and magnesium were determined in mice at the end of the 14-week experimental diet. No significant differences were noted in the serum concentrations of these components (Table 2). In contrast, significant changes were noted in calcium, phosphorus, and magnesium concentrations in the urine of mice after feeding with magnesium. Specifically, over 24-fold increase in calcium concentration and > 85% reduction in the phosphorus concentration were noted in *asj* mice fed high magnesium diet (Table 2, groups C and E). In addition, the concentration of urinary magnesium was significantly increased in *asj* mice fed high magnesium diet (Table 2, groups C and E). Feeding mice with elevated magnesium content resulted in more calcium and magnesium excreted into urine, suggesting direct interactions by which magnesium and calcium ions compete in phosphorus binding leading to more soluble magnesium phosphate complexes.

**Table 2:** Calcium, phosphorus, and magnesium concentrations in the serum and urine of mice^1)^

Group	Serum concentration			Urine concentration		
	Calcium (mg/dL)	Phosphorus (mg/dL)	Magnesium (mg/dL)	Calcium (mg/dL)	Phosphorus (mg/dL)	Magnesium (mg/dL)
A	10.8 ± 0.2	6.5 ± 0.4	3.5 ± 0.1	3.4 ± 0.7	89.9 ± 18.0	43.9 ± 3.4
B	10.6 ± 0.1	6.7 ± 0.6	4.3 ± 0.4	4.1 ± 0.6	116.1 ± 13.0	51.9 ± 2.3
C	10.3 ± 0.4	5.2 ± 0.6	4.8 ± 0.3	99.7 ± 16.6**	16.4 ± 3.2**	61.6 ± 0.3**
D	10.9 ± 0.2	6.2 ± 0.3	4.1 ± 0.2	4.5 ± 0.5	141.1 ± 14.4	46.0 ± 1.9
E	10.9 ± 0.1	7.2 ± 0.4	4.5 ± 0.1	103.7 ± 18.4**	9.0 ± 1.4**	61.5 ± 0.2**

^1)^ Data are expressed as means ± SE; n = 8-13 mice per group. ^*^ *P* < 0.01, as compared with *asj* mice on control diet in group B. For description of different groups, see Table 1.

### High magnesium content in the diet does not cause hormonal and metabolic alterations

As inhibition of ectopic mineralization by magnesium could involve changes in the parathyroid hormone (PTH) action [18], we also measured the serum concentrations of PTH by an ELISA assay in mice fed control diet or diet enriched in magnesium at the end of the experimental diet. Although individual variability in PTH values was noted, the average concentrations were not statistically different in different groups (Table 3).

**Table 3:** Measurements of serum PTH and urinary albumin*1)*

Group	Serum PTH (pg/mL)	Urine albumin (g/dL)
A	56.8 ± 8.7	< 0.01
B	44.2 ± 7.6	< 0.01
C	47.0 ± 13.3	< 0.01
D	61.6 ± 9.3	< 0.01
E	38.9 ± 4.7	< 0.01

^1)^ Data are expressed as means ± SE; *n* = 8-13 mice per group. No statistical differences are noted as compred to the *asj* mice on control diet in group B. PTH, parathyroid hormone. The urinary albumin levels are below the detection limit of 0.01 g/dL. For description of different groups, see Table 1.

To determine whether feeding with magnesium affects renal function, we measured urinary albumin concentrations as an indicator of renal function. In all mice analyzed, albumin levels in the urine samples were below the detection limit of 0.01 g/dL (Table 3), suggesting normal renal function in mice fed with magnesium for up to 14 weeks of time.

## DISCUSSION

Diseases of ectopic mineralization of connective tissues range from rare heritable diseases, such as GACI, to common maladies in the ageing population, such as arteriosclerosis. The characteristic feature of GACI is profound arterial mineralization often diagnosed by prenatal ultrasound during pregnancy. The affected children are born with cardiovascular complications with older reports indicating ~85% mortality rate at 6 months of age [19]. Current medical strategies preventing or reversing the connective tissue mineralization present in GACI are ineffective and novel experimental therapeutics are clearly needed. One study reported that intensive treatment with bisphosphonates lowered mortality of GACI to 55% at 6 months [8], while other studies showed very little, if any, effect [20]. Bisphosphonates have also been reported to be accompanied by severe side effects, particularly on the development of bones [14]. The potential efficacy of bisphosphonates has been also explored in *asj* mutant mouse as a preclinical platform for GACI. The results demonstrated that bisphosphonate treatment may be beneficial by a dual effect for preventing ectopic soft tissue mineralization while correcting decreased bone mineralization in *asj* mice [21]. Recently, it was shown that recombinant ENPP1-Fc fusion enzyme, when administered subcutaneously in *asj* mice with reduced ENPP1 enzymatic activity, prevents mortality and vascular mineralization [22].

There are several lines of evidence suggesting that diet, particularly with respect to its magnesium content, may modify the severity of ectopic mineralization. Early studies using transgenic mice with targeted ablation of the *Abcc6* gene as a model for PXE have demonstrated that increased levels of magnesium in the diet, five times over the standard rodent diet, completely abolished the ectopic mineralization in these mice [16, 17]. At the same time, addition of phosphate, two times over the standard diet, when combined with reduced (20%) magnesium content, significantly accelerated the mineralization in mouse models for PXE and GACI; this diet has been designated as the “acceleration diet” [15, 23, 24]. In addition, maternal “acceleration diet” during pregnancy can influence the degree of ectopic mineralization in the offspring [25].

The results of our study clearly demonstrate that magnesium, when added to the mouse diet in amounts that increase the magnesium concentration by five-fold, is able to prevent the ectopic mineralization in skin and vascular tissues in *asj* mice when the mothers are also placed on the same diet during pregnancy. When only the *asj* mice were kept on high magnesium diet post-weaning, or only the mothers were placed on high magnesium diet while pups were maintained on control diet, the efficacy of magnesium on preventing mineralization is reduced. Supplementation of magnesium in the mouse diet did not change serum calcium, phosphorus, and magnesium levels, while increased calcium and magnesium concentrations were found in the urine. No overt side effects were noted in the mice fed with five-fold increase of magnesium content in mouse diet.

The mechanisms for the inhibition of ectopic mineralization by magnesium could be systemic interfering with the calcium metabolism mediated through the PTH synthesis and/or secretion. In support of this suggestion are observations that magnesium deficiency is associated with insufficient PTH action and can lead to reduced responses to calcitropic hormones [18]. However, determination of serum PTH levels in the treated *asj* mice did not reveal significant differences. An alternate mechanism may involve direct interactions between magnesium and calcium ions in the mineralization process, *i.e*., magnesium competes calcium-phosphate binding and forms magnesium phosphate complexes which are soluble, thereby preventing the mineral deposition of calcium phosphate complexes. The elevated urinary calcium and magnesium results favor the latter hypothesis.

Our observations have implications for the clinical management of patients with GACI caused by *ENPP1* mutations. Specifically, the results of our study suggest that dietary magnesium might be helpful for treatment of patients with GACI. Identification of mutations in the *ENPP1* gene can be used for confirmation of the clinical diagnosis, carrier detection and presymptomatic identification of affected individuals with family history of GACI. Prenatal genetic testing from chorionic villus sampling or from fetal cells in maternal circulation is also possible for families with affected individuals. For patients with family history, as soon as the clinical diagnosis and mutation analysis have been made during pregnancy, elevated magnesium in maternal diet might slow down the progression of GACI in the fetus and improve the quality of postnatal life of patients with this, currently intractable, disease. The results from the preclinical mouse model of GACI suggest that magnesium supplementation in pregnant mothers or the newborn child alone would not be sufficient, but rather the pregnant mothers would have to receive treatment followed by continued treatment of the affected newborn suspected of GACI. It should be noted that the daily dose of magnesium administered to the mice was higher than the recommended daily dose for humans (300-400 mg per day). Thus, the efficacy in humans should be tested in controlled clinical trials with careful monitoring of side effects. The similar clinical features and pathophysiology in GACI and other common conditions presenting with vascular mineralization suggests that these disorders may be treated by a common approach—dietary magnesium treatment.

## MATERIALS AND METHODS

### Mice and diet

C57BL/6J-*Enpp1^asj^*/GrsrJ mice on a C57BL/6J background were obtained from The Jackson Laboratory (Bar Harbor, ME); these mice are referred to as the *asj* mice [15]. The wild type and homozygous *asj* mice were generated from heterozygous breedings. The mice were maintained on standard rodent laboratory diet (Laboratory Autoclavable Rodent Diet 5010; PMI Nutritional International, Brentwood, MO) under standard conditions. Mice were divided into five groups (A, B, C, D and E) based on their *Enpp1* genotype and treatment regiments. Groups A and B were wild type and *asj* mice, respectively, fed a control diet for both pregnant mothers and pups until pups became 14 weeks of age. The pregnant mothers in group C were fed the control diet during pregnancy and nursing. At 4 weeks of weaning, the *asj* pups were placed on diet with a five-fold increase in magnesium for another 10 weeks. The pregnant mothers in group D were fed the high magnesium diet during pregnancy and nursing. At 4 weeks of weaning, the *asj* pups were placed on control diet for another 10 weeks. The *asj* mice in group E and their corresponding mothers received the high magnesium diet during pregnancy and postnatally. At 14 weeks of age, the mice were sacrificed for analysis. For different groups, see Table 1.

All protocols were approved by the Institutional Animal Care and Use Committee of Thomas Jefferson University.

### Histopathology of soft connective tissues

Biopsies from muzzle skin containing vibrissae (left side) as well as the internal organs (left kidney and descending thoracic aorta) were fixed in 10% phosphate-buffered formalin and embedded in paraffin. Paraffin sections (6 μm) were stained with hematoxylin-eosin (H&E) and Alizarin red stains using standard methods [15].

### Quantification of calcium and phosphorus

To quantify the calcium content in mouse tissues, muzzle skin biopsies (right side), right kidney, and abdominal aorta were harvested and decalcified with in HCl solution. The solubilized calcium was determined in the HCl supernatants colorimetrically by the ơ-cresolphthalein complexone method (Calcium (CPC) Liquicolor; Stanbio Laboratory, Boerne, TX). The values for calcium were normalized to tissue weight. Calcium in the serum and urine samples was quantitatively assayed as above. The serum and urinary phosphorus content was determined with a Malachite Green Phosphate Assay kit (BioAssay Systems, Hayward, CA).

### Quantification of magnesium

The magnesium concentrations in the mouse serum and urine were measured using the QuantiChrom^TM^ Magnesium Assay Kit (BioAssay Systems). The magnesium contents in the HCl supernatants of tissues were also measured using this kit.

### Energy dispersive X-ray analysis

Paraffin sections of muzzle skin were mounted onto carbon carrier and imaged using energy dispersive X-ray analysis and topographic mapping [26]. The elemental composition was analyzed with a FEI 600 Quanta FEG scanning electron microscope (FEI Company, Eindhoven, The Netherlands) fitted with an Octane Super SDD EDS detector (EDAX, Sandy, UT). X-ray topographic maps of calcium and phosphorus were acquired using Spirit software version 1.07.05 (Princeton Gamma-Tech, Rocky Hill, NJ).

### Serum parathyroid hormone and urinary albumin assays

Serum PTH concentrations were measured using a Mouse PTH 1-84 ELISA Kit (Immutopics Inc., San Clemente, CA). Urinary albumin concentrations were determined using a QuantiChrom^TM^ BCG Albumin Assay Kit (BioAssay Systems).

### Statistical analysis

The results in different groups of mice were evaluated by Student's two-tailed *t*-test. Statistical significance was reached with *P* < 0.05. Analyses were conducted using SAS 9.4 (SAS Institute, Cary, NC).

## Abbreviations

GACI: generalized arterial calcification of infancy
PXE: pseudoxanthoma elasticum
WT: wild type
*asj*: ages with stiffened joints
